# Stakeholder perceptions of supporting patients’ return to work in primary care: a qualitative study

**DOI:** 10.3399/BJGPO.2024.0280

**Published:** 2025-12-19

**Authors:** Rosie Harrison, Gwenllian Wynne Jones, Vaughan Parsons, Ira Madan, Carolyn A Chew-Graham, John Pemberton, Gemma Mansell, Karen Walker-Bone, Nadine E Foster, Benjamin Saunders

**Affiliations:** 1 School of Medicine, Keele University, Keele, UK; 2 Faculty of Life Sciences and Medicine, King's College London, London, UK; 3 Occupational Health Service, Guy’s and St Thomas' NHS Foundation Trust, London, UK; 4 Aston Institute of Health and Neurodevelopment, Aston University, Birmingham, UK; 5 Monash Centre for Occupational & Environmental Health, Monash University, Monash, Australia; 6 UQ Clinical Trials Centre, University of Queensland, Brisbane, Australia

**Keywords:** Qualitative research, patient perspectives, occupational health, return to work, primary health care

## Abstract

**Background:**

Around 2.5 million people in the UK are absent from work due to ill health, yet, for many, accessing work-orientated vocational support (VS) to facilitate return to work (RTW) is challenging. The majority of fit notes are issued in primary care, making this an ideal setting to provide VS.

**Aim:**

As part of the Work And Vocational advicE (WAVE) randomised controlled trial (RCT), we explored the delivery of VS by trained vocational support workers (VSWs), from the perspectives of patients, VSWs, employers, and GPs.

**Design & setting:**

In the WAVE RCT, patients from 10 UK general practices were randomised to the offer of usual care or usual care plus VS. This qualitative study explored stakeholder perspectives of the VS intervention.

**Method:**

Semi-structured interviews were conducted with participants in the intervention arm (*n* = 10), employers, VSWs, and GPs (*n* = 5). Interviews were audio-recorded, transcribed, and analysed using thematic analysis. Public and patient involvement and engagement was embedded throughout.

**Results:**

Taking a person-centred, individualised approach to VS enabled VSWs to identify and mitigate RTW obstacles and support participants’ self-efficacy to proactively negotiate RTW. The perceived independence of the VSWs from employers and health care was considered important and facilitated more open discussions about capabilities and RTW planning.

**Conclusion:**

Findings indicated that individualised and independent VS offered to patients referred from primary care was perceived by all stakeholders to be valuable to patients absent from work due to illness and supported their RTW planning. These insights can inform future models of VS.

## How this fits in

The detrimental economic and social effects of people absent from work due to ill health are well known and facilitating people’s return to the workforce is a key policy priority, yet accessing vocational support (VS) is challenging for many people, and there is a paucity of evidence about what makes effective VS from the perspectives of different stakeholders. In this study, we interviewed people receiving VS, their employers, vocational support workers (VSWs), and healthcare professionals to understand their perspectives on effective VS provision. Our findings showed that effective VS took an individualised, person-centred approach to return to work (RTW) planning, which encompassed honest discussions of the person’s capabilities and potential adaptions to their job, facilitated by the perceived independence of the VSW. Primary care remains the first point of contact for people absent from work due to ill health and is therefore an ideal setting to provide effective VS from a VSW.

## Introduction

The global socioeconomic burden of employee ill health through absence and presenteeism is well established.^1^ Around 2.5 million people in the UK are economically inactive due to long-term sickness,^2^ a rise of 500 000 since 2019.^3^ While this is partly due to the ongoing economic and social impacts of the COVID-19 pandemic, it may also be influenced by an uncertain and rapidly changing working environment and employment context.

Work participation benefits people’s overall health and wellbeing,^4^ yet many people who are absent from work experience difficulties in building and sustaining the self-efficacy necessary to overcome obstacles to RTW.^5^ Vocational rehabilitation can help to increase people’s self-efficacy,^6^ however there is limited access to VS in the UK. Only around half of employees have access to occupational health services,^7^ leaving the majority to seek occupational support from healthcare professionals (HCPs) whose role is often limited to the issue of ‘Statement of Fitness for Work for Social Security or Statutory Sick Pay’, known as ‘fit notes’. Fit notes provide employers with a clinical judgement on whether a person is medically fit for work or not. HCPs can also indicate that the individual ‘may be fit’ for work if the employer enacts suggested amendments to their role and/or duties.^8^ While fit notes can be issued by a wide range of HCPs, the majority are issued in primary care. However, a lack of time, capacity, and training presents challenges to having effective conversations about work in healthcare encounters.^9^ It is therefore important that people are directed to appropriate, evidence-based VS to facilitate their RTW.

The Work And Vocational advicE (WAVE) randomised controlled trial (RCT) tested the effectiveness of adding a brief VS intervention to usual primary care for patients who receive a ‘fit note’ from their general practice.^10^ Two trained VSWs, from occupational support and counselling backgrounds, worked with patients in the intervention arm to identify obstacles to working and developed a tailored RTW plan to mitigate these barriers, as described elsewhere.^11^

Key to the successful uptake of any health intervention is how it is perceived and experienced by stakeholders involved in its delivery and receipt.^12^ This study reported a qualitative study nested in the WAVE RCT exploring perceptions and experiences of delivering and receiving the VS intervention, from the perspective of patients, VSWs, GPs, and employers/line-managers.

## Method

One-to-one semi-structured interviews were conducted with participants in the WAVE RCT intervention arm, as well as VSWs, employers, and GPs.

This article conformed to standard qualitative reporting guidelines.^13^

### Recruitment and sampling

Intervention arm participants who consented to further contact were purposively sampled at the 6-week follow-up point in the trial for variation in key characteristics of age, sex, reason for the ‘fit note’, and RTW status, and mailed an interview invitation and information sheet. All but one patient participant had had contact with a VSW at time of interview. While the original intention was to only interview participants who had received the VS intervention, it only became apparent during the interview that this patient from the intervention arm of the RCT had not accessed the service. However, they had experience of being recruited to the trial and were enthusiastic about sharing their views, and it was therefore decided to include them within the sample. Interview participants were asked for permission to contact their employer to invite them to a separate interview.

GPs from participating practices were invited by email, followed by a telephone call or further email. The two VSWs delivering the intervention were also invited to an interview.

### Data collection

Individual interviews were conducted between November 2022 and July 2023, via telephone (*n* = 4, audio-recorded) and Microsoft Teams (*n* = 11, video-recorded). Participants provided verbal, audio/video-recorded consent at the beginning of their interviews, which was reaffirmed at the end.

Thirteen interviews were conducted by the lead author (RH, female) and two by co-author (BS, male) (both PhD, with social science background and experienced in qualitative research), who were not previously known to the participants. While we aimed to reach sufficient data saturation, the point at which further data no longer offer new insights,^14^ recruitment challenges meant that this was not possible for all participant groups.

Separate topic guides were used with the four participant groups (see Supplementary Boxes S2–S5), which were developed with the patient and public involvement and engagement (PPIE) group, but were not pilot-tested, and were iteratively refined with early findings informing later interviews. Field notes were not taken during interviews, so as not to impact on rapport-building.

### Data analysis

Interview recordings were professionally transcribed, checked, and anonymised by RH. An inductive thematic analysis approach^15^ was used to explore how the intervention was perceived by the different participant groups and to identify any similarities and differences of perspective within and between groups, facilitated by the qualitative software program NVivo (version 12). Following this approach, a sample of early transcripts was independently coded by RH and BS and a coding framework agreed upon was used in subsequent coding. Coded data were analysed to develop categories and themes that were then discussed at team meetings, including with public co-investigator, JP. Analysis was iterative, with emergent findings used to further refine topic guides for subsequent interviews. To prevent overburdening participants, transcripts were not returned to participants for comment, nor did participants provide feedback on the findings.

Data from each group (patients, VSWs, GPs, and employers/line-managers) were analysed separately; findings from each dataset were then mapped onto one another to explore similarities and differences. Linked patient–employer interviews were compared for similarities and differences.

### PPIE

PPIE members were involved throughout the WAVE trial, informing the research question and qualitative study design. During meetings, they supported the development of patient information sheets, recruitment materials, and topic guides. Public co-investigator JP also contributed to the qualitative data analysis.

## Results

### Interview participant characteristics

Forty-three patient participants were invited (see Supplementary Box S1), with 10 interviews conducted. Five were male and five female, aged 28−64 years, and based in three regions of England: South London (*n* = 3), West Midlands (*n* = 5), Wessex (*n* = 2). The two participants still on sick leave were waiting for hospital appointments delaying their RTW.

Reasons for their fit note were musculoskeletal conditions (MSK) (*n* = 3), mental health issues (*n* = 3), and other physical health conditions (*n* = 4). No participants withdrew from the interview study. Table 1 outlines the patient participant characteristics.

**Table 1.** Patient participant characteristics

ID	Sex	Age, years	Fit note reason	RTW status at time of interview	Type of job
P01	F	25–29	Other	RTW	Social care
P02	F	35–39	Other	Sick leave	IT
P03	M	60–64	Other	Medical retirement	Civil servant
P04	M	55–59	MSK	Medical retirement	Emergency services
P05	M	35–39	MSK	Sick leave	Education
P06	F	50–54	MSK	RTW	Emergency services
P07	M	60–64	Other	RTW	Warehouse operator
P08	M	40–44	Mental health	Unemployed	Warehouse operator
P09	F	45–49	Mental health	RTW	Management role
P10	F	40–44	Mental health	RTW	Transport

F = female. M = male. MSK = musculoskeletal. RTW = return to work.

Interviews were conducted with both VSWs delivering VS in the trial. Five participants consented to their employer/line-manager being invited and two line-managers were interviewed, from a social care setting and IT company, respectively. HCPs from 10 general practices were invited, but only one female GP, with 10 years’ experience in practice, was interviewed.

Interviews with patients ranged from 30–60 minutes. Employer and GP interviews ranged from 27–34 minutes, and VSW interviews ranged from 55–90 minutes.

Two main themes were identified, as outlined in Table 2

**Table 2.** Main themes and sub-themes identified

Theme	Sub-themes
The value of VS provided	Participant–VSW contact
	Individualised support
	VSW independence
Perceptions of the employer’s role in RTW planning	Employee–employer contact
	Potential for VSW–employer contact

VS = vocational support. VSW = vocational support worker.

### Theme 1: The value of VS provided

#### Participant–VSW contact

Due to COVID-19 restrictions at the time, all VS was delivered remotely via telephone, which all patient participants reported as a convenient method of support. The protocol^10^ did not pre-define the duration of support offered, and therefore the number and frequency of sessions were negotiated between the participant and VSW. To facilitate RTW, sessions focused on developing an individualised RTW plan, which participants reported resulted in a ‘natural’ conclusion to the support:

*'We had regular contact up until the point where we said we’ve come to a natural end now because I’d gone back to work*.*'* (P10, female [F])

Not having a fixed time frame for VS also alleviated any ‘pressure’ participants might feel regarding their progress:

*'If you’re told an end date then you kind of feel pressure to make sure things are right by then. I wouldn’t want to go over that date then because you feel like you have to definitely stop at that date. So, I think leaving it open is the right thing to do*.*'* (P01, F)

This open-ended approach also enabled VSWs to support patients with more complex health conditions, who may require longer-term contact. However, the VSWs found this model challenging as they had previously worked within a fixed timeframe or set number of sessions:

*'If you're working in an employee assistance programme, you get paid for six sessions, those sessions are done, that’s it. So, I felt like some pressure from within myself and some misunderstanding. "Really I needed to have got these participants through and back to work." And of course, that comes back to long-term health conditions. That doesn't fit that model.'* (VSW01)

All participant groups reported a valuable aspect of the intervention concerned discussing an appropriate time frame for RTW. VSWs reported that they encouraged participants to realistically and honestly assess their capabilities when thinking about this time frame, as a premature return was perceived to be detrimental for the individual and their relationship with colleagues if they could not adequately perform their job role:

*'I have a participant at the moment who has had COVID twice. Learning from previous experience, feeling that they wanted to go back to work and went back to work too soon, and how de-skilled, demoralised they felt because they weren't able to manage.'* (VSW01)

Participants reported that discussing an appropriate timescale for RTW with the VSW supported this decision making and prevented a premature RTW:

*'I probably would’ve gone back sort of soon after it happened, and I knew that would’ve been the wrong thing to do, but seeing how short-staffed everyone is. So, just having an extra voice saying*, *"No, actually, you need to take time off for yourself, and get yourself better," that all helped*.*'* (P06, F)

Employers reported similar concerns about the timing of their employee’s RTW, and highlighted the perceived benefit of the VS intervention in leading to their employee’s sustained RTW:

*'I was really worried that she’d come back too quickly, and she’d be off sick again. That was my very biggest concern, so I didn’t want to overload her, to begin with. But no, she’s had no time off at all, since she came back, so to me that’s, whatever it* [the intervention] *did, it worked.'* (Employer [E]01)

#### Individualised support

Taking an open-ended approach to the duration of support enabled VSWs to deliver a more individualised approach in addressing RTW obstacles faced by participants. All participant groups saw this as important, as people may experience similar health conditions differently, and a participant’s individual obstacles to RTW may not be fully captured in their fit note:

*'It could be more holistic in a sense because there was lots of other things that were maybe having an effect. Rather than just the one issue they may have been off sick, it may say their fit note one reason but then the actual underlying problems could be more widespread and open*.*'* (VSW02)

Similarly, participants highlighted underlying, non-work-related difficulties that needed to be addressed:

*'People very quickly think that things are work-related, often there’s another issue that you’re not addressing that maybe you didn’t know you’ve got*.*'* (P09, F)

Taking this holistic approach to discussing barriers to RTW helped VSWs support patients in their RTW decision making, and patients indicated that this helped to build their self-efficacy by prompting them to assess their capabilities and any possible role adaptions that would facilitate RTW:

*'*[The VSW helped develop] *the concrete plan of what I might like to ask for and what am I allowed to ask for and encouraging me to think about being creative with those proposals, what parts of my job … was I thinking about yet. I think I can do "that" but I really don’t think I can do "this" so kind of using my skills and experience creatively and kind of … presenting it in a way that was going to be mutually beneficial. And yeah that’s not something I’ve ever really had to do before. I … just thought, this is my job, this is what I’m contracted to do. And so I’m either going to be doing that or I’m not going to be working for them anymore.'* (P02, F)

VSWs could also support patients’ sustained RTW by promoting their self-efficacy to problem-solve any issues encountered during early RTW:

*'It was looking at giving people options as well so if they’re back at work and it’s not quite working out, what could change and what could stop them going off work again. Just trying to open up … ask lots of open questions almost to get them to come up with the solution themselves and then kind of put that in a way that they could kind of put that into practice*.*'* (VSW02)

As reported in Table 1, not all interview participants had achieved RTW at time of interview, with two patients experiencing medical retirement and one unemployment because of their health condition. The two participants who were still on sick leave were waiting for secondary care input before they could RTW. While this was an external barrier to RTW that was beyond the remit of VS, the VSW supported patients in preparing for their RTW while they waited, as described later.

#### VSW independence

All participant groups highlighted the importance of VSWs being perceived as independent from their employer, the Department of Work and Pensions, and healthcare providers. VSWs considered their independence to be an important foundation for establishing a trusting relationship with the participant:

*'I did take on board that it was important to say to everybody, just to reiterate, “I have no relationship with your employer. I'm employed by Keele University. This is my role … This doesn't affect any* [state] *benefits that you might be receiving”* … *Again, I guess that’s about encouraging trust and just clarifying who you're employed by and what your role is.'* (VSW01)

It was also proposed that VSWs’ perceived independence could facilitate a more open discussion of RTW planning and coordination, as patients may not feel obliged to ‘prove’ their illness is genuine in the same way as when talking to their GP, where these discussions are perceived to have implications:

*'I thought* [the intervention] *was really good because people were given the time, had good, practical information in more time than you can go through it and it was as an independent person, so they’re not trying to prove how ill they are to us. I think sometimes the dynamic here can be different.'* (GP01)

Talking to an independent professional enabled participants to discuss how they felt about their health and work situation, particularly in the context of RTW planning, without fear of potential negative consequences:

*'It was like somebody I could speak to away from home or work. Somebody totally outside of the bubble, if you like, about anything.'* (P04, male)

### Theme 2: Perceptions of the employer’s role in RTW planning

#### Employee–employer contact

Patient participants who had limited or no contact with their workplace during their sickness absence reported that this lack of contact negatively impacted their approach to RTW planning. However, they reported that the VSW supported them to initiate contact with their employer; for instance, through creating an action plan, that gave patients some accountability for taking proactive steps in RTW planning, which they viewed positively:

*'So, we had like an action plan timetable thing that we’d put in like a challenge set for me and then when I had to do it by and what I’d do. Like just stuff like getting in contact with my manager because there had been no contacts. So just to make that first step and then to create a meeting.* […] *We created a plan to follow basically and then once it was on there I knew I had to do it.'* (P01, F)

To promote patients’ self-efficacy, VSWs reported that they encouraged patients to plan support sessions around their action plan to provide the opportunity to review their progress:

*'I could kind of like I said "advise you from the shadows" and you could take that and kind of do it and then we could catch up before and after. Help plan, I could help sort of build that plan on how you are going to do things and then you could go ahead and do that*.*'* (VSW02)

Patients also reported that developing an RTW plan provided reassurance that they could negotiate their RTW with their employer, and gave them the confidence to adapt these plans as necessary. In response to the question, *'And how useful did you find that actual concrete planning?'*, one participant answered:

*'Yeah very useful, I mean essential in fact because I mean it’s literally been sat in my notebook now for literally months. But I know that if I get a call this afternoon* [from employer], *"oh yeah, can we arrange this meeting and we’ll have it on this day at this time, in two days’ time" that I’ve just got that plan there. And because it has been so long it might not be exactly what I’d like to say but I’ve got that there now, it’s really taken a lot of stress out of what that meeting might even feel like, you know*.*'* (P02, F)

VSWs reported supporting patients with the emotional aspects of RTW planning and coordination through building the patient’s confidence and RTW self-efficacy regarding workplace policies and procedures:

*'People genuinely are empowered and do feel able to*, *"I think I might contact HR," or*, *"Yes, I might chase that up," or*, *"Yes, actually, I haven't looked at my staff handbook. I will have a look at what’s required of my employer and me." Yeah, so those glimmers of light where someone might say, "Oh, yes, I hadn’t thought about that. There is something I could actually take some control over."'* (VSW01)

Patients reported feeling reassured and empowered to take a proactive approach to RTW:

*'It helped me to have that "can do" attitude, helped reassure me that my thought processes were along the right lines*.*'* (P10, F)

#### Potential for VSW–employer contact

For patients who were experiencing significant barriers to RTW, there was the option for direct contact between VSWs and the participants’ employer/line-manager.^10^ While this contact did not take place for any of the patients, some patient participants expressed reservations about the hypothetical prospect of VSWs directly contacting their employer:

*'I don’t think work need to know everything about you. I’d gone back to work, that’s really all they need to know.'* (P10, F)

However, others reported feeling reassured that the VSW could contact their employer as a *‘back up’* if necessary:

*'It* [contacting employer] *was something I thought I had to do myself, really. But it was good to have that back up there. So, if it went all pear-shaped for me, I could have her input with work*.*'* (P01, F)

VSWs were generally positive about the possibility of directly contacting employers, but reported feeling this may undermine their aim of promoting the participants’ self-efficacy to proactively engage with the employer:

*'I would have welcomed the opportunity to explore* [direct contact], *whilst also feeling, "Well, I feel this is the participant’s place," and encouraging that self-efficacy.’* (VSW01)

The two line-managers interviewed were positive about the possibility of contact with VSWs and saw the benefit of the intervention to both employees and line-managers, particularly in supporting line-managers who are less experienced in dealing with employee work absence:

*'Because some of the things that you kind of just do without thinking* [as a manager] *are because of experience ultimately. And I think some people just need that little bit more of a guide and our HR team is great but … any other support that can be given I think is always well received*.*'* (E02)

## Discussion

### Summary

This study explored the delivery of a VS intervention from the perspectives of patients, employers, VSWs, and GPs. Similarities across the participant groups included the perceived benefits of an individualised, person-centred approach to VS that can identify individual obstacles to RTW and promote people’s self-efficacy to navigate their workplace sickness absence and RTW procedures and policies. Discussions around the timescale for RTW were perceived to be an important aspect of effective VS, as RTW before the patient is ready was viewed by participants as counterproductive to achieving sustained RTW. The perceived independence of the VSWs from employers and HCPs was considered conducive to the provision of effective VS, as it helped facilitate more open, honest conversations about an individual’s capabilities.

Differences across the participant groups included perceptions of the duration of contact between patients and VSWs. Patient participants were positive about receiving VS until a workable RTW plan had been developed; however, VSWs pointed to the challenges associated with taking an open-ended approach rather than offering a fixed number of sessions. While VSWs and employers saw potential benefits in VSWs directly contacting employers to support RTW planning, patients were more pessimistic about this option.

### Strengths and limitations

A strength is the inclusion of the views of different stakeholders, including those delivering and receiving VS, and patients’ employers/line-managers. The multidisciplinary team involved in data analysis was also a strength, including expertise from health and work research, occupational and environmental health, social science, and academic general practice, increasing the trustworthiness of the findings.

A limitation is that, due to recruitment challenges, we were unable to reach sufficient data saturation across all participant groups. While half the patients agreed for their employer to be contacted, only two employers were interviewed. It may be that these employers were more positive about the intervention than those who did not respond, thus favourably influencing the findings. Lead GPs or practice managers from the 10 general practices involved in the trial were asked to circulate the invitation to GPs in their practice on multiple occasions over a 12-month period, yet only one GP interview was completed. It may be that these stakeholders’ lack of direct involvement in the trial resulted in their lack of engagement in the qualitative study, as they may not have viewed the study as being relevant to them. Additionally, some practice managers cited workload issues and lack of time as a reason for GPs being unable to participate, which may reflect the current stretched capacity in UK general practice. Had a greater number of GPs and employers been recruited this would have likely given rise to additional insights on the delivery of VS in this context.

When interpreting the findings, it is important to acknowledge the influence of the researcher on participants’ interview responses. However, a reflexive approach was taken throughout, with the interviewers attending to and acknowledging any underlying preconceptions. Participants were made aware that the researchers conducting interviews were part of the WAVE trial team and not clinicians, and were interested in understanding both positive and negative aspects of their experiences.

### Comparison with existing literature

Comparison can be drawn with our previous research where we carried out a discourse analysis of appointments in which VS was delivered^11^ as part of a feasibility trial prior to the main WAVE RCT. While similarities can be drawn, the present findings provide new insights not identified in our previous research; notably, the perceived value of VSWs being seen as independent, facilitating a trusting relationship.

Similarities can also be drawn with a recent UK-based consensus groups study that identified patients’ priorities for RTW outcomes,^4^ including assessing capacity and readiness to RTW and collaboration between key stakeholders. Our findings showed close alignment with these priorities and identified the importance to patients of receiving individualised, person-centred support that incorporates considerations of an appropriate RTW time frame. These new insights reflect NHS principles around personalised care and support planning^16^ and NICE guidelines on individualised care,^17^ and illustrate how person-centred VS can be delivered.

A recent narrative review of RTW after chronic disease^18^ highlighted the importance of the employer–employee dynamic on subjective and objective evaluations of the person–environment fit and therefore RTW outcomes. Similarly, a recent interview study^19^ exploring RTW barriers with those on long-term sick leave in Norway identified that the subjective experiences of the employer and employee were the most influential on RTW outcomes. This importance of the subjective aspects of RTW planning was reflected in our findings, which also illustrated the perceived value for patients of receiving VSW support to prepare for effective formal contact with their employer, through the creation of RTW plans and support navigating workplace procedures.

Similarities can also be drawn with research exploring the disclosure of health information to employers. A recent UK interview study exploring the perspectives of employers and people with chronic pain around RTW planning^20^ found that while employers advocated early disclosure of health conditions, most people with chronic pain were reluctant to do this due to potentially adverse consequences. Our findings reflected these differing views from employers and employees about the disclosure of health conditions and identified the importance of the VSW in supporting these conversations through providing patients with independent advice and facilitating honest evaluations of their health and capabilities.

### Implications for practice

The role of GPs in sickness certification is currently being debated, with the Department of Work and Pensions’ 2022 decision to expand the issue of fit notes to nurses and allied HCPs and the recent call-for-evidence for ‘fit note reform’.^21^ Yet, primary care remains the first point of contact for most individuals and is therefore ideally positioned to provide early supportive conversations. The ongoing pressure and workload demand on GPs and other HCPs means capacity for engaging in work conversations may be limited.^9^ Our findings pointed to the potential benefits of VSWs, delivering individualised VS to patients identified within primary care, to facilitate their RTW.

Other policy initiatives aim to improve access to VS through the integration of employment advisors into MSK pathways^22^ and the introduction of local WorkWell Vanguard Partnerships.^23^ Our findings highlighted the importance of VS being perceived by patients as independent from their employer, HCPs, or government policy, and this may be an important consideration for these initiatives.

Furthermore, the findings highlighted the need for work-orientated VS to be person-centred, as the RTW journey is an individualised and often complex process. It is important that professionals delivering VS have the necessary knowledge, skills, and training to tailor support to patients’ individual needs in a holistic manner, as outlined in NICE guidelines.^17^ The findings we have presented can influence policy initiatives as well as the design of future VS interventions aiming to address the needs of people with a wide range of health conditions.

## Data

The dataset relied on in this article is available from the corresponding author on reasonable request.
